# Amelanotic malignant melanoma in a child^☆^

**DOI:** 10.1016/j.abd.2022.09.017

**Published:** 2023-12-06

**Authors:** Anwei Chen, Faliang Ren

**Affiliations:** Department of Dermatology, Chongqing Medical University Affiliated Children’s Hospital, Chongqing, China

Dear Editor,

A 3-year-old girl presented with a 7-month history of a pinkish nodule on the left leg. The lesion was firstly about 2 mm papule, then it enlarged in size within 7 months, and erosion and exudation of the nodule appeared under frequent scratching. The girl had no systemic symptoms, and her family history was unremarkable. The ulcerated nodule had no response to oral antibiotics. Physical examination showed a pinkish nodule 2.5 × 2.5 cm in diameter on the inner of the left leg, with a hard texture, irregular border, and ulcerated surface, closely surrounded by two scattered small papules (Fig. 1A). Blood tests including routine blood tests, liver and kidney functions, and coagulation results were normal. According to the clinical manifestations, dermatofibrosarcoma protuberans, sporotrichosis, and atypical mycobacterial infections were considered clinically. The nodule was completely excised under an operation and a histopathological examination of the total lesion was consequently performed. Histopathologic findings (Fig. 1B‒D, Fig. 2A‒B) demonstrated pseudoepitheliomatous hyperplasia and ulcerated lesion, melanocyte proliferation with angulated nuclei in a lentiginous pattern in dermal-epidermal junction, a few atypical melanocytes with pagetoid scatter in the epidermis and epithelioid melanocytes partially in nests in the upper dermal component. In the lower bottom field of the lesion, poor circumscription, dense cellularity, lack of maturation, and infiltrative growth were detected. High magnification exhibited hyperchromatic nuclei, large irregular nuclei with prominent atypia and poor differentiation, nuclear molding in the dermal component, and tumor cells extended invasively into the deep tissues. Immunohistochemical staining (IHC) (Fig. 2C‒D, Fig. 3A‒D) showed Melan A (+), S-100 (+), HMB-45 (−), P16 (−), Cyclin D1 (+), Ki-67 (about 20%, +) suggesting elevated proliferation. The diagnosis of amelanotic malignant melanoma (AMM) was confirmed based on the ulcerated irregular nodular, microscopic finding, and IHC results. Investigations, including computed tomography of the head and chest, and ultrasonography of superficial lymph nodes, liver, spleen, and kidney were normal. These results did not indicate any lesioned metastasis. The patient is carefully being followed-up, for up to 2-years, and she is still luckily alive and in healthy condition with normal growth and development.

**Figure 1:**
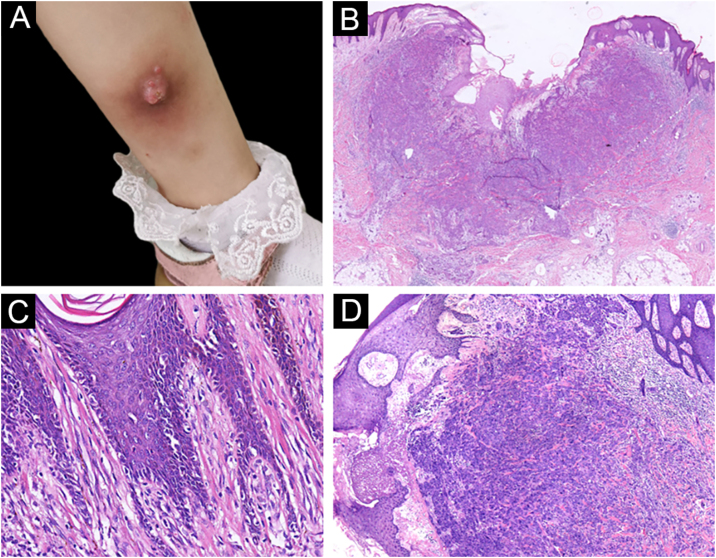
(A) A pinkish nodule with an ulcerated surface on the inner of the left leg. (B) Ulcerated lesion, and infiltrative growth (Hematoxylin & eosina, ×10). (C) Melanocyte proliferation with angulated nuclei in a lentiginous pattern (Hematoxylin & eosina, ×200). (D) Pseudoepitheliomatous hyperplasia and epithelioid melanocytes partially in nests in the upper dermal component (Hematoxylin & eosina, ×40).

**Figure 2:**
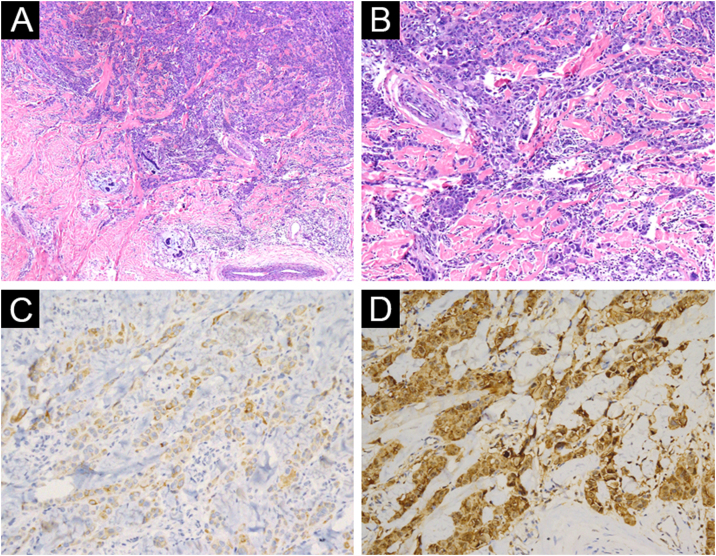
(A) Poor circumscription, dense cellularity, lack of maturation, and infiltrative growth were detected in low bottom (Hematoxylin & eosina, ×40). (B) High magnification exhibited hyperchromatic nuclei, large irregular nuclear with prominent atypia, and extended invasively into the deep tissues (Hematoxylin & eosina, ×100). (C) Melan A (+), ×200. (D) S100 (+), ×200.

**Figure 3:**
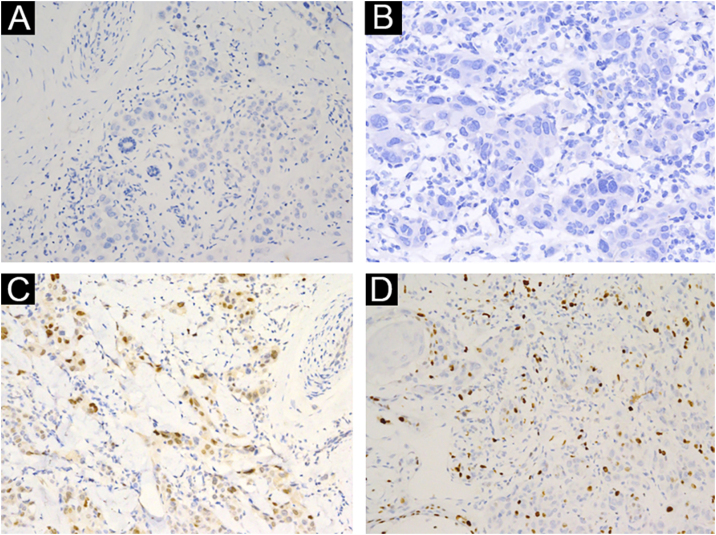
(A) HMB-45 (−), ×200. (B) P16 (−), ×200. (C) Cyclin D1 (+), ×200. (D) Ki-67 (about 20%, +), ×200.

AMM is a rare type of melanoma and is challenging to diagnose for lacking the classic clinical and pathological features of malignant melanoma (MM), especially for children.^1^ The common locations of AMM for younger children are the head, neck, and extremities, while there are very few literature available indicating that AMM in childhood involves unusual areas, such as iris, vola, and intra-cranium, and may be a complication of oculocutaneous albinism and neurocutaneous melanosis.^2^, ^3^, ^4^ Although pathological result directly ruled out the infectious disease, AMM can clinically or pathologically present as spitzoid lesion, which is easily misdiagnosed as Spitz nevus and leads to inappropriate or delayed treatment. The key to distinguishing the AMM and Spitz nevus is based on the comprehensive analysis of clinical presentations and histopathologic changes. Clinically, the lesion is often no bigger than 1 cm in diameter and vanishingly ulcerates. Under microscopy, melanocyte maturation, clear demarcation, good symmetry, clefts, and Kamino bodies are detected in Spitz nevus. Absent these changes of Spitz nevus, lack of maturation, poor differentiation, large irregular nuclei with prominent atypia, high proliferation index and mitoses in the bottom of the tumor, are identified in AMM. In most cases, histopathology can give the correct diagnosis, but in some cases, it may be misleading or hard to make a different diagnosis, immunohistochemical markers can help us better understand the nature of lesions. In our case, the negativity for P16 and positivity for CyclinD1 are consistent with the diagnosis of melanoma, reversibly in Spitz nevus. In this fact, we propose that physicians keep vigilant for amelanotic nodules in children. Molecule testing for melanoma emerges to help convey the diagnosis, such as BRAF V600E, NRAS, ROS, TERT-p, and NTRK by pyrosequencing.^5^ Surgery is the most effective way for MM. Adjuvant therapy such as TNF-α injection in children remains unclear.

## Financial support

None declared.

## Authors’ contributions

Anwei Chen: Wrote the manuscript.

Faliang Ren: Wrote the manuscript, provided the case and revised the manuscript.

## Conflicts of interest

None declared.
